# Early-Life Stress Triggers Juvenile Zebra Finches to Switch Social Learning Strategies

**DOI:** 10.1016/j.cub.2015.06.071

**Published:** 2015-08-17

**Authors:** Damien R. Farine, Karen A. Spencer, Neeltje J. Boogert

**Affiliations:** 1 Edward Grey Institute of Field Ornithology, Department of Zoology, University of Oxford, Oxford OX1 3PS, UK; 2 Department of Anthropology, University of California, Davis, Davis, CA 95616, USA; 3 Smithsonian Tropical Research Institute, Panamá 0843-03092, Panama; 4 School of Psychology and Neuroscience, University of St. Andrews, St. Andrews KY16 9JP, UK; 5 Department of Zoology, University of Cambridge, Cambridge CB2 3EJ, UK

## Abstract

Stress during early life can cause disease and cognitive impairment in humans and non-humans alike [1]. However, stress and other environmental factors can also program developmental pathways [2, 3]. We investigate whether differential exposure to developmental stress can drive divergent social learning strategies [4, 5] between siblings. In many species, juveniles acquire essential foraging skills by copying others: they can copy peers (horizontal social learning), learn from their parents (vertical social learning), or learn from other adults (oblique social learning) [6]. However, whether juveniles’ learning strategies are condition dependent largely remains a mystery. We found that juvenile zebra finches living in flocks socially learned novel foraging skills exclusively from adults. By experimentally manipulating developmental stress, we further show that social learning targets are phenotypically plastic. While control juveniles learned foraging skills from their parents, their siblings, exposed as nestlings to experimentally elevated stress hormone levels, learned exclusively from unrelated adults. Thus, early-life conditions triggered individuals to switch strategies from vertical to oblique social learning. This switch could arise from stress-induced differences in developmental rate, cognitive and physical state, or the use of stress as an environmental cue. Acquisition of alternative social learning strategies may impact juveniles’ fit to their environment and ultimately change their developmental trajectories.

## Highlights

• Juvenile zebra finches learn foraging skills from their parents
• Stress hormone exposure triggers juveniles to learn from unrelated adults instead
• Stress may be a cue juveniles use to inform their behavioral strategies
• Switching social learning strategy may alter developmental trajectories adaptively

## Teaser

Farine et al. demonstrate that young birds learn foraging skills from their parents. However, early-life exposure to experimentally elevated stress hormones prompts juveniles to switch strategies and learn only from unrelated adults. This may help them to compensate for their poor natal environment and acquire more adaptive behaviors in nature.

## Results and Discussion

Social learning, where animals learn from observing or interacting with others, enables traditions to be transmitted across generations [4]. Social structure can greatly affect information spread [7–9] and the transmission of novel behaviors [10–13], while individuals’ position within their social network can alter their fitness [14–16]. However, it is unclear whether individuals’ characteristics modulate information transmission through social networks: do individuals pay equal attention to all their associates? If not, what strategies do they use to decide who to learn from [5], and how are these influenced by the environment, both past and present?

One major determinant of individual variation in social behavior, and potentially social learning, is exposure to stress in early life [17]. Developmental stress has been linked to variation in dispersal distance [18], patterns of social contacts [17], and information use [19]. We hypothesize that developmental stress could also guide social learning strategies, in terms of who to copy when faced with novel environmental challenges. Here we investigate whether (1) individuals of the highly gregarious zebra finch (*Taeniopygia guttata*) are biased in whom they learn from and (2) juveniles exposed to experimentally elevated stress hormone levels in early life later adjust their learning strategies. Zebra finches use social learning to acquire their songs and song preferences [20], when choosing mates [21], and when deciding where and what to eat [22]. Here we focus on the social acquisition of foraging skills.

To determine how developmental stress affects social learning strategies, we exposed half of the chicks in each of 13 broods to physiologically relevant doses of the avian stress hormone corticosterone (CORT) on days 12–28 post-hatching. Once chicks reached nutritional independence at ∼35 days, we released six to seven families into each of two identical aviaries (N = 29 and 34 finches, respectively). This resembles flock composition in the wild, where neighboring families forage together for food (unpublished data). For 20 days, we collected a complete record of all birds’ foraging associations from passive integrated transponder (PIT) tags fitted to each bird and detected by radio-frequency identification (RFID) antennae fitted to two feeders in each aviary. We then introduced a novel foraging task [23, 24] on day 21 and measured each individual’s latency to first approach and to first solve (see the Experimental Procedures for details and Table S1 for descriptive statistics). Of the 63 birds, 39 solved the task. These solvers represented 11 of the 26 adults and 28 of the 37 juveniles. Half of the 28 juvenile solvers were controls, and half were treated with CORT.

### Individuals Copy Adults to Acquire Novel Foraging Skills

We quantified social information transmission in each aviary by combining the 20-day social foraging network with the birds’ task-solving latencies in a network-based diffusion analysis (NBDA) [25, 26]. NBDA quantifies the rates at which individuals acquire information or novel traits following a previously measured social network. It estimates how much individuals’ social learning rates are accelerated, or the likelihood of learning a novel task solution increased, when their associates demonstrate this new information (represented by the parameter *s*; see the Supplemental Experimental Procedures). NBDA has generated significant insights into how animals acquire social information about novel food locations and foraging behaviors [7, 9–12, 27]. NBDA of a single task solution cannot distinguish between imitation and socially facilitated learning (i.e., demonstrators attract naive associates to the task, where the latter then learn asocially). However, here we are interested in social learning strategies in terms of who learns (either directly or indirectly) from whom, rather than in the social learning mechanism involved.

**Figure 1:**
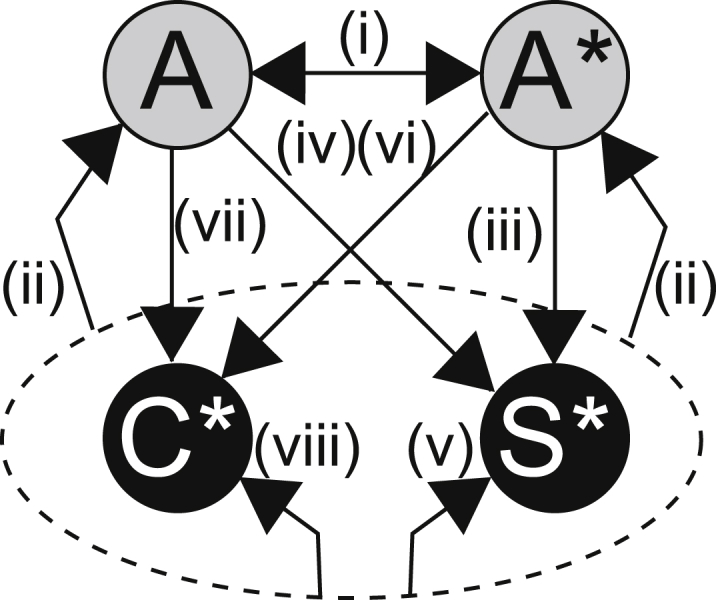
Summary of Edge Classifications The full network was partitioned into eight different networks, each containing a different class of edge (see the main text), and different combinations of these were used in an information-theoretic framework to evaluate our hypotheses (see also Figure S1). Gray nodes (A) are adults; black nodes are juveniles, split into control (C) and CORT/developmentally stressed (S) treatments. The ^∗^ represents individuals from the same family (thus, here one adult is a parent and the other is unrelated). Edges from all juveniles (dashed oval) represent edges from related and unrelated juveniles combined (both C and S treatments and all unrelated juveniles are included).

We used a recently developed variant [9] of NBDA that quantifies transmission rates between different types of social network connections (“edges”). Instead of estimating a single social transmission rate between all types of individuals (regular NBDA), we partitioned the edges into eight separate directed networks (Figure 1) containing all the incoming edges from adults to adults (i), juveniles to their parents and unrelated adults (ii), parents to their CORT-treated offspring (iii), adults to unrelated CORT-treated juveniles (iv), control and CORT juveniles to CORT juveniles (v), parents to their control offspring (vi), adults to unrelated control juveniles (vii), and control and CORT juveniles to control juveniles (viii). The sum of these eight networks is the observed network (i.e., no edges occurred in more than one network). For each network, we estimated a separate rate of social transmission *s* for each category of connections. For example, if juveniles learned exclusively from each other, then we would expect a high *s* for the juvenile to juvenile network and *s* = 0 for other networks.

We used an information-theoretic approach, constructing all possible models and comparing these to evaluate our hypotheses (see the Supplemental Experimental Procedures and Figure S1). We used corrected Akaike’s information criterion to allow for model uncertainty, summing up the Akaike weights to calculate the level of support for each hypothesis (following [28]). Analysis of the data from both diffusions (one in each aviary) revealed that information spread through the foraging association networks (supported by 99.7% of model weights; Table 1) rather than through homogeneous networks (i.e., where all associations are set to 1; supported by 0.3% of model weights), or via asocial learning. There was also evidence (10.7% of model weights) for a 2.5% faster learning rate in the second aviary. Table S2 contains the top five models, accounting for >98% of model weights.

We estimated the relative importance of three major pathways of information transmission: (1) individuals learned from everyone, (2) individuals learned exclusively from adults, and (3) individuals learned exclusively from juveniles (Figure S1). Models containing transmission from adults only were best supported (total Akaike weight = 81.7%; Table 1), suggesting that both adults and juveniles learned almost exclusively from adults. These results provide some of the strongest empirical support yet for “directed social learning” [29] in a naturalistic, family-structured social context. This is consistent with the notion that individuals should tailor their strategies to acquire relevant traits. Similarly, primates tend to copy higher-ranking, i.e., nominally more successful, conspecifics [30–32].

### Developmental Stress Modulates Juveniles’ Social Learning Strategies

We then tested whether social learning rates were the same across each network (same *s*) or differed in each network (different *s*; see Figure S1). We found strong evidence for a different *s* for each network (total Akaike weight = 99.3%; Table 2) and for differences in social learning strategies among juveniles. Using the Akaike weights for each model, we obtained the model-averaged estimates for each *s* (Table 3). Rates of transmission (*s*) differed between control and CORT-treated juveniles. Control juveniles relied more on their parents (*s* = 9.9) than on unrelated adults (*s* = 6.6) to learn the novel foraging skill. That is, one unit of social network connection to a knowledgeable parent increased control juveniles’ likelihood of learning the behavior by one-third compared to a unit of social network connection to a knowledgeable but unrelated adult. In contrast, the model-averaged rate of information transmission from parents to CORT-treated juveniles was very low (*s* = 0.005). This is despite CORT-treated and control juveniles having similar foraging association strengths to parents (mean = 0.31 and 0.32, respectively). Instead, CORT-treated juveniles learned almost exclusively from unrelated adults (*s* = 5.7). Relative transmission rates were similar in the two aviaries (Table S3). These results suggest that increased exposure to stress hormones during post-natal development resulted in a switch by juveniles from vertical to oblique social learning strategies. The extent to which this switch was driven by active model choice by juveniles, rather than a parental decision to be more tolerant toward their control offspring, is an interesting question for future research, although we never observed adult aggression toward juveniles.

### Social Learning Rates Are State Dependent

Naive adults and juveniles varied in their latencies to solve the task and their reliance on social information. Naive adults were slower than juveniles at *approaching* the task (linear mixed-effects model [LMM] of task approach latencies [all models herein include “family” nested within “aviary” as random effects]: estimate ± SE = 3684.31 ± 1662.66, *t*_41_ = 2.22, p = 0.032), but *solved* the task faster (LMM of solve latency: estimate ± SE = −7370.60 ± 3115.71, *t*_25_ = −2.37, p = 0.026). However, once the task was being demonstrated, every unit of social network connection to a knowledgeable adult increased adults’ learning rate by only 2.5 times. Naive juveniles were at least twice as likely as naive adults to acquire the behavior from adult demonstrators: their social learning rate increased by at least 5.7 times the baseline rate per unit increase in network connection to knowledgeable adults. Higher rates of social learning in juveniles as compared to adults have also been reported in blue tits (*Cyanistes caeruleus*) [33], great tits (*Parus major*) [11], and white-throated magpies (*Calocitta formosa*) [34].

Because information about the novel foraging task was transmitted socially, who learned was largely dependent on which adults demonstrated the task and the propensity of juveniles to forage with these demonstrators. Control and CORT-treated juveniles learned from unrelated adults at similar rates (Table 3), suggesting that they did not differ in their *ability* to acquire the trait socially when using the same category of demonstrators. However, CORT-treated juveniles started to solve the task sooner than control juveniles (LMM of solve latency: estimate ± SE = −7603.62 ± 3250.04, *t*_14_ = −2.34, p = 0.035). Thus, even though control juveniles learned rapidly from their parents, whereas CORT-treated juveniles did not, CORT-treated juveniles still acquired the trait sooner. This could be because they relied more on individual trial-and-error learning [35]. Alternatively, CORT-treated juveniles may have simply had access to information about the task sooner by associating with more unrelated adults (mean network association strength of 1.62 versus 1.57 for control juveniles), who made up the majority of (potentially demonstrating) adults in each aviary.

### Conclusions

The social network guided the transmission of a novel foraging task solution through flocks of birds, but not all connections had an equal likelihood of transmitting information. Importantly, despite both relying on social learning from adults when acquiring the novel foraging skill, CORT-treated and control juveniles differed in their social learning strategies. Control juveniles largely copied their parents to acquire the novel foraging skill. CORT-treated juveniles, in contrast, relied on learning from unrelated adults only.

Developmental stress may induce switches in social learning strategies in various ways. These may involve changes in developmental rate [36], stress responsiveness [36], or cognitive and social skills [1]. However, these cannot completely explain why CORT-treated juveniles did not acquire the novel foraging task solution from their parents, despite associating with them almost as strongly as did control juveniles in the social foraging network. Theory suggests that developmental stress may be used as an informative cue about an individual’s environment [2, 37], which could range from parental investment to natal habitat quality. If so, it may enable juveniles to avoid becoming trapped in a negative feedback loop provided by a bad start in life, by programming them to adopt alternative, and potentially more adaptive, behaviors.

## Experimental Procedures

### Rearing and Hormone Treatment

We individually housed 13 domesticated zebra finch pairs and synchronized the within-brood hatching dates of their eggs by replacing them with plastic dummies until the brood was complete. Chicks were individually marked, and approximately half in each brood were randomly assigned to the following experimental CORT treatment [19]: they were fed 20 μl of CORT (Sigma Aldrich; 0.155 mg/ml in peanut oil) twice daily, giving a total dose of 6.2 μg CORT/day. This dose is known to result in plasma CORT levels comparable to those naturally induced in untreated chicks exposed to an acute stressor [36]. Control chicks were fed 20 μl of pure peanut oil when their siblings received CORT. Experiments were conducted under Home Office Animals (Scientific) Procedures Act project license no. 60/4068 and personal license no. 60/13491.

### Free-Flying Aviaries

When chicks were 37 ± 1 days old, we fitted them and their parents with PIT tags attached to unique color rings and released families together into one of two identical aviaries (3 × 3.1 × 3.2 m) on the same day. Aviaries contained seven (N = 34 birds) and six families (N = 29 birds), respectively, and both were equipped with two identical transparent feeders containing finch seed at all times, except during the novel foraging task experiment (see below). Feeders were fitted with RFID antennae to record the PIT tags of zebra finches as they freely entered and exited the feeders.

### Inferring the Social Network

The data loggers attached to the RFID antennae provided a complete record of individuals visiting the feeders simultaneously. From this temporal data stream, we extracted bouts of foraging activity using a well-established algorithm [38, 39] and used the simple ratio index to calculate association strengths (see the Supplemental Experimental Procedures) with the asnipe package [40] in R.

### Novel Foraging Tasks

On the mornings of days 21–23, we removed feeders at 9:00 a.m. and, after 1 hr of food deprivation, presented a novel foraging task on a platform (1 × 1 × 1 m) in each aviary. This task consisted of four white plastic foraging grids (8 × 12 × 2 cm), each containing 12 wells (2-cm diameter, 1.5-cm depth; 48 wells in total). Each well contained spinach (0.5 × 0.5 cm) covered with a lid. Lids consisted of yellow cardboard squares (2 × 2 cm) with upward-folded corners and felt bumpers (2-cm diameter, 0.5-cm height). The same baited grids had been presented for 2 days preceding the experiment, and four lids were added on top of each grid (but not covering the wells) 1 day before the experiment. This habituated birds to the novel objects and prevented neophobia from inhibiting skill acquisition.

The zebra finches were left to discover how to remove the lids from the wells to obtain the food reward, which we filmed from different angles. We returned to the aviaries each hour to re-bait the grids, for a total of three 1-hr trials per day over 3 days. At the end of each test day, foraging grids were removed from the aviary and the regular feeders were returned. From the videos, we scored the latency (counted in seconds from the start of the experiment, excluding times when the task was being re-baited/not presented) of each bird’s first approach within pecking distance of a lid, as well as their first and all subsequent task solutions. A bird was considered to have solved the task when it deliberately lifted the lid completely out of the well (i.e., not accidentally kicking/knocking it off) so that it could access the spinach underneath. We identified individuals from the videos using their unique color rings. We considered the latency of each bird’s first task solve to be the time point at which it switched from a naive to an informed state. Table S1 contains task performance statistics. We then used these latencies to model the spread of this information through the zebra finch flocks using NBDA. Full details and model specifications are provided in the Supplemental Experimental Procedures.

## Author Contributions

N.J.B. and K.A.S. devised the experiment, N.J.B. collected the data, D.R.F. performed the analysis, D.R.F. and N.J.B. drafted the manuscript, and all authors contributed to revisions. D.R.F. and N.J.B. are equal contributors.

## Figures and Tables

**Table 1:** Relative Importance of Three Major Pathways of Information Transfer

Network	All Conspecifics	Adults Only	Juveniles Only	Total
Association	17.7	81.7	0.3	99.7
Homogeneous	<0.01	<0.01	0.3	0.3
Total	17.7	81.7	0.6	

Summary of the total Akaike weight (%) for all models testing the hypotheses that individuals learned the novel foraging task solution from all classes of conspecifics, individuals learned it exclusively from adults, and individuals learned it exclusively from juveniles. Models were all additive (see Table S4 for weights from multiplicative models). Networks therein were either foraging association informed or homogeneous (see the Supplemental Experimental Procedures). Support for asocial models was 3.69 × 10^−21^.

**Table 2:** Relative Support for Uniform versus Varying Rates of Transmission across Different Networks

Network	Same *s*	Different *s*	Total
Association	0.4	99.3	99.7
Homogeneous	0.3	0.04	0.3
Total	0.7	99.3	

Summary of the total Akaike weight (%) for all models testing the hypotheses that *s* was the same across all networks in each model or *s* differed across all networks in each model. Models were all additive (see Table S5 for weights from multiplicative models). Networks therein were either foraging association informed or homogeneous (see the Supplemental Experimental Procedures). Support for asocial models was 3.69 × 10^−21^.

**Table 3:** Model-Averaged Estimates of Information Transmission Rates between Classes of Individual

Network	Edges From	Edges To	Social Learning Rate (*s*)	Upper 95% CI	Lower 95% CI
i	adults	adults	2.22	5.08	0.32
ii	CORT and control juveniles	adults	0.006	0.07	0
iii	parents	CORT-treated juveniles	0.005	0.08	0
iv	unrelated adults	CORT-treated juveniles	5.75	10.88	2.29
v	CORT and control juveniles	CORT-treated juveniles	0.004	0.10	0
vi	parents	control juveniles	9.86	18.26	6.29
vii	unrelated adults	control juveniles	6.62	11.71	2.08
viii	CORT and control juveniles	control juveniles	0.13	0.25	0

Each network contained the directed social network links from individuals of a given class (e.g., parents) to another class (e.g., offspring). This approach provides social learning rate estimates per unit of social network connection to knowledgeable individuals for each class independently (given by *s* in the models; see the Supplemental Experimental Procedures). CI, confidence interval. See also Table S3.

## Supplemental Information

Document S1. Supplemental Experimental Procedures, Figure S1, and Tables S1–S5

Document S2. Article plus Supplemental Information
