# Supplementary material for: Early-Life Stress Triggers Juvenile Zebra Finches to Switch Social Learning Strategies
Source: Curr Biol. 2015 Aug 17;25(16):2184–8. doi: 10.1016/j.cub.2015.06.071 (PMC4540255; doi:10.1016/j.cub.2015.06.071)
Supplement: Document S1. Supplemental Experimental Procedures, Figure S1, and Tables S1–S5 [file mmc1.pdf]

Current Biology
Supplemental Information

Early-Life Stress Triggers Juvenile Zebra Finches to Switch Social Learning Strategies

Damien R. Farine, Karen A. Spencer, and Neeltje J. Boogert

Figure S1: Framework used to partition the different categories of edges in the observed foraging network to evaluate two hypotheses: 1) the major pathways of information transmission: (a) individuals learnt from everyone (all eight networks were included, i.e. the sum of these networks equals the foraging network), (b) individuals learnt exclusively from adults, where *s* for networks with edges originating from juveniles (i.e. networks (ii), (v) and (viii)) was constrained to 0, or (c) individuals learnt exclusively from juveniles, where *s* for networks with edges originating from adults (i.e. networks (i), (iii), (iv), (vi), (vii)), was constrained to 0, and 2) whether *s* was the same or different in each network. Networks are described in Figure 1. Here, A (red nodes) are adults, and juveniles (blue nodes inside dashed oval) are partitioned into control chicks (C) and stressed (CORT) chicks (S). Individuals marked with a * are members of the same family (thus, one adult is a parent and the other is unrelated in this diagram).

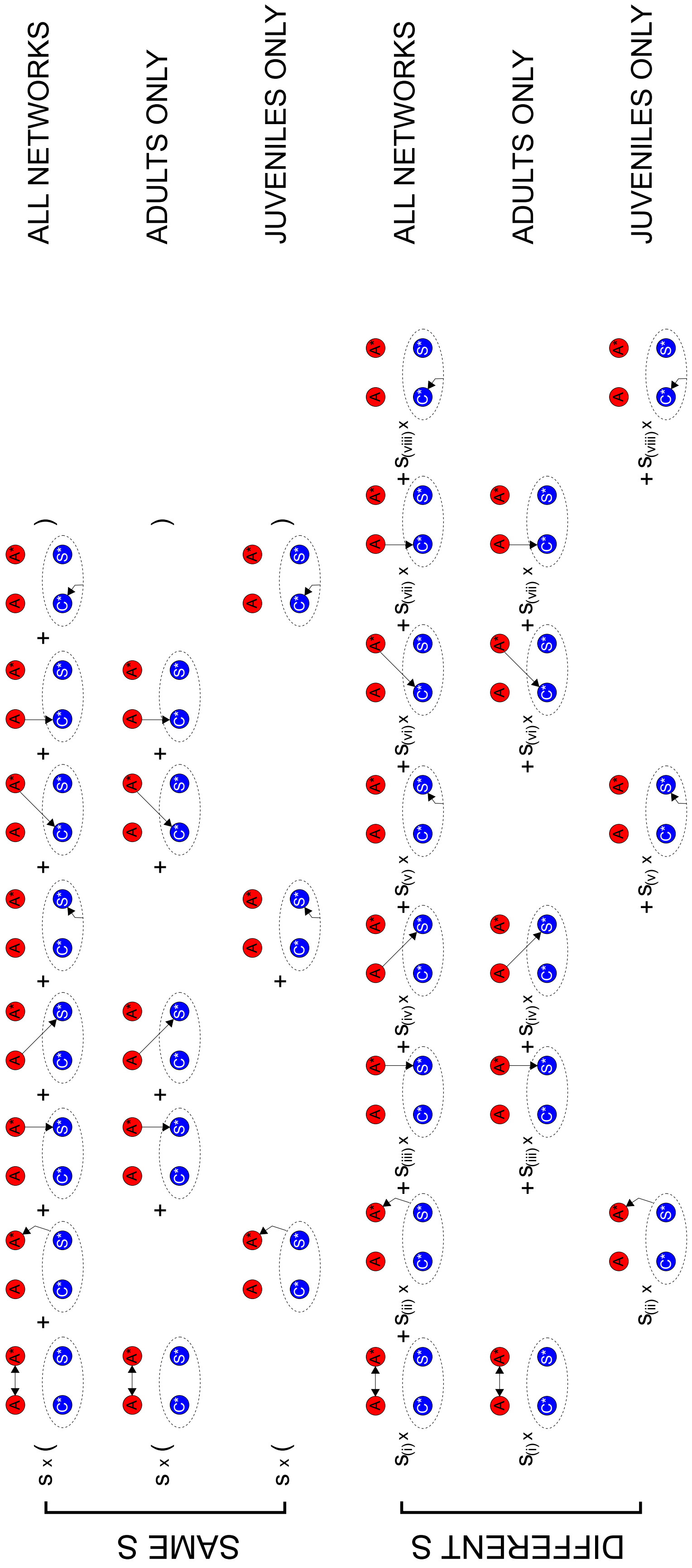

Table S1: Descriptive statistics for approach and task solving latencies in seconds across the two aviaries

	Latency to approach task			Latency to solve task		
	**Adults (*n*=19)**	**Control juveniles (*n*=16)**	**CORT juveniles (*n*=20)**	**Adults (*n*=11)**	**Control juveniles (*n*=14)**	**CORT juveniles (*n*=14)**
n in aviary 1	9	8	11	7	8	8
n in aviary 2	10	8	9	4	6	6
Mean	11328	7758	6743	8534	20205	12602
Median	7837	7464	4624	7828	21107	9807
Range	28814	15069	28363	28827	31694	27571
Min	2327	2509	2352	2339	2584	2566
Max	31141	17578	30715	31166	34278	30137

Table S2: Top 5 models ranked by Akaike weight. The best-supported models had a mixture of constant and non-constant asocial learning rates (i.e. decreasing over time, but the rate of decrease was small at only 0.75), all were additive, and all included the foraging-association informed rather than a homogeneous social network. Subsequent models had an Akaike weight < 1%.

**Model rank**	**Incoming edges from**	***s* different or same across networks**	**Baseline**	**Aviary difference**	**df**	**AICc**	**Akaike weight (%)**
1	Adults only	Different	Non-constant	NA	8	898.48	54.2
2	All	Different	Constant	NA	10	900.78	17.2
3	Adults only	Different	Constant	NA	7	900.79	17.1
4	Adults only	Different	Non-constant	0.62	9	901.99	9.4
5	Adults only	Different	Constant	2.64	8	906.80	0.8

Table S3: Estimates of information transmission rates (*s*) between classes of individual using the top model (Table S2) and run for each aviary separately. Each network fit an *s* parameter to the social network links from individuals of a given class (e.g. parents) to another class (e.g. offspring). The top model constrained *s* parameters for edges from juveniles (marked by a *) to 0 (i.e. the model was an 'adults only' model).

**Network**	**Edges from:**	**Edges to:**	**Aviary 1**	**Aviary 2**
i	Adults	Adults	1.6	2.8
ii	CORT & control juveniles	Adults	0*	0*
iii	Parents	CORT-treated juveniles	0	0
iv	Unrelated adults	CORT-treated juveniles	3.1	6.7
v	CORT & control juveniles	CORT-treated juveniles	0*	0*
vi	Parents	Control juveniles	9.7	12.1
vii	Unrelated adults	Control juveniles	2.95	8.6
viii	CORT & control juveniles	Control juveniles	0*	0*

Table S4: Summary of the total Akaike weight (%) for all *multiplicative* models testing the hypotheses that (i) individuals learnt the novel foraging task solution from all classes of conspecifics, (ii) individuals learnt it exclusively from adults, and (iii) individuals learnt it exclusively from juveniles. Networks therein were either foraging association-informed or homogeneous (see Supplemental Experimental Procedures). Support for asocial models was 3.69x10-21.

**Network**	**(i) All conspecifics**	**(ii) Adults only**	**(iii) Juveniles only**	***Total***
Association	6.3x10-8	1.6x10-7	1.6x10-5	*7.1x10-5*
Homogeneous	8.0x10-8	5.3x10-8	5.3x10-6	*7.4x10-5*
**Total**	1.4x10-7	**2.1x10-7**	**2.1x10-5**	

Table S5: Summary of the total Akaike weight (%) for all *multiplicative* models testing the hypotheses that (i) *s* is the same across all networks in each model, or (ii) *s* differs across all networks in each model. Networks therein were either foraging association-informed or homogeneous (see Supplemental Experimental Procedures). Support for asocial models was 3.69x10-21.

**Network**	**(i) *S* same**	**(ii) *S* different**	***Total***
Association	4.7x10-5	2.4x10-5	*7.1x10-5*
Homogeneous	5.8x10-5	1.7x10-5	*7.4x10-5*
**Total**	**1.0x10-4**	**4.1x10-5**	

SUPPLEMENTAL EXPERIMENTAL PROCEDURES

Inferring the social network

We constructed the social network using the following method: (i) individuals were individually fitted with unique (10-digit hexadecimal code) electronic Passive Integrated Transponder (PIT) tags attached to a plastic, uniquely coloured, leg ring. (ii) Tags were recorded by RFID antennae integrated into the feeder entrances and the bird identity (PIT tag code), feeder identity, time and date of each feeder visit were saved onto the internal memory of the PIT-tag reader devices (Dorset ID, The Netherlands). (iii) Foraging groups were identified by extracting peaks of feeder visit activity in the temporal data stream using a clustering algorithm (i.e. a Gaussian-mixed model [S1, S2]). On average, such foraging groups spent 290 seconds at the feeder, and contained 7 individuals. (iv) The association strength (edge weights in the network) between each dyad of birds in each aviary was defined as the rate of feeding co-occurrence, calculated as the number of observations of both individuals in the same foraging group divided by the number of observations of at least one individual in a foraging group (the simple ratio index which ranges from 0: never observed together, to 1: always observed together). While observing two individuals in the same foraging group may not necessarily be meaningful for a single event, this approach provides highly robust estimates of foraging associations through repeated observations of dyads, in this case 4216 group observations in aviary 1 and 4077 group observations in aviary 2. This also represents a complete record of every single visit to each feeder by all individuals in each aviary over the 20 observation days. (v) We created a single network combining the data from all 20 days of observation, calculated using the asnipe library [S3] in R.

Network-based diffusion analysis (NBDA)

In the standard NBDA model [S4-S6], the rate at which an individual adopts a new trait is estimated as a function of its connections to knowledgeable individuals ("demonstrators"). The effect of these demonstrators is scaled by the parameter *s*, which is the rate of transmission per unit of social network connection to demonstrators. The multi-NBDA model builds on this by including multiple candidate networks, each of which can have a different value for *s* (cf different rates of transmission through different networks) [S7]. We partitioned the zebra finch foraging association network into eight networks (given in the main text, see also Table 3), each representing a sub-network of the complete foraging network from each aviary and containing a single class of edges (see Figure S1). Each network contained the full set of individuals, where those that were not connected to any edges of the given class (for example adults in a juvenile-to-juvenile network) were isolates (i.e. not connected to anyone). We also tested asocial learning models (i.e. no edges in the network) and models of undifferentiated or "homogeneous" social transfer (i.e. fully-connected network where every edge = 1). We used the continuous variant of multi-NBDA, and included either a constant or varying (i.e. becoming faster or slower) baseline rate of transmission over time. We combined the data from the two aviaries when estimating parameter values (using each aviary's network with its diffusion data), and included "aviary" as an asocial parameter to account for potential differences in transmission rates between them. We used model-averaging to evaluate different hypotheses and estimate transmission rates through each network. The parameters for each model were fitted using the optim function in R.

Equations

We used a recently-developed variant of network-based diffusion analysis (NBDA) that allows transmission rates to be quantified across multiple candidate networks [S7]. The functional form of this model for M networks is given by:

$$\lambda_i(t) = \lambda_0(t)\left(\sum_{k=1}^{M}\left[s_k\sum_{j=1}^{N}a_{ijk}z_j(t)\right]+1\right)\left(1-z_i(t)\right)$$

where $\lambda_0(t)$ is the baseline rate of acquisition (herein referred to as asocial learning when $s =$ 0), s_k is the rate of social transmission through the edges given by network k, a_{ijk} is the edge weight between individuals i and j in network k (all networks had the same N individuals, but all $a_{ij} = 0$ for nodes that were not connected to edges represented in network k, such as adults in networks v and viii (see Table 3)), and $z_i(t)$ is the status of individual i at time t (0 = naive, 1 = informed). Because diffusions occurred in different aviaries, we also included an asocial variable $LP_i = \beta x_i$, where x_i is an indicator variable showing whether individual i was in aviary 2 ($x_i = 1$) or aviary 1 ($x_i = 0$), to account for potential differences in baseline rates of learning between the two aviaries. If models containing this asocial variable are better supported than models not containing it (see below), then it suggests that individuals' learning rates varied between the two aviaries. The estimated parameter value gives the differences in rates (i.e. difference in intercept of the baseline or asocial learning rate). We also included both additive and multiplicative models in our model-averaging procedure. Additive models assume that individuals acquire the novel trait as a direct consequence of observation, while multiplicative models assume that social learning occurs indirectly, e.g. by knowledgeable individuals attracting naïve ones to the novel foraging task, where the latter then learn from their own experience. Multiplicative models therefore use the aviary asocial parameter as a variation both

in the intercept of the learning rate over time as well as the slope due to the increase in social learning over time across the two aviaries. Our models are given by the following equations:

$$\lambda_i(t) = \lambda_0(t)\left(\sum_{k=1}^{M}\left[s_k \sum_{j=1}^{N} a_{ijk} z_j(t)\right] + exp(LP_i)\right)\big(1 - z_i(t)\big)$$

for additive, and:

$$\lambda_i(t) = \lambda_0(t)\left(\sum_{k=1}^{M}\left[s_k \sum_{j=1}^{N} a_{ijk} z_j(t)\right] + 1\right) exp(LP_i)\big(1 - z_i(t)\big)$$

for multiplicative models, where $exp(LP_i)$ is set to 0 in models without the asocial parameter included (see *model averaging* below). Because we found very little support for multiplicative models (<0.01% of the Akaike weight), we only included the results of these in the supplemental results (Tables S4 & S5).

We also included models testing for asocial-only learning (all edges in all networks were set to 0) and for undifferentiated social learning (all edges in all networks were set to 1). When testing hypotheses, such as whether individuals learnt only from adults and/or from juveniles, we summed the Akaike weights of all models in each category. All analyses were conducted in R, and model parameters were fitted to the data using the optim function.

Model averaging

We used model averaging to answer two questions: 1) Which are the major pathways of information transfer? and 2) Does information transmission differ through different social ties? We first constructed every possible model with all combinations of the following factors:

- Social transmission model (additive or multiplicative)
- Baseline, or asocial, learning rate (constant or non-constant)
- Network type (foraging-association or homogeneous)
- Asocial parameters (effect of aviary or no asocial parameter)
- Constrained rates (transmission from all individuals, from adults only, or from juveniles only) (used to evaluate question 1)
- Varying transmission rates (same *s* or different *s*) (used to evaluate question 2)

This resulted in 96 unique models, each of which was fitted to the data. In addition, we included 4 models of asocial transmission (with variants for additive/multiplicative, constant/non-constant), in which all *s* values were constrained to 0.

Using log-likelihood of the fit of the model to the data, we calculated the AIC values for each model, and ranked models based on the AICc values. From the resulting Δ AIC values, we then calculated the relative support ($e^{-0.5*\Delta AIC}$). The Akaike weight for that model was the relative support divided by the sum of the relative support for all models.

To measure the Akaike support for each hypothesis, we summed the Akaike weights for all models containing that hypothesis. For example, the support for foraging association versus homogeneous networks is the sum of the weight of the models where the input networks were the observed foraging associations, divided by the total sum of Akaike weights (shown as the

row sums in Table 1). In Figure S1, we show the different models used to evaluate question 1 (all, adult only, or juvenile only networks) and question 2 (same *s* vs different *s*). These 6 combinations were fit with each combination of social transmission model (additive vs multiplicative), baseline learning rate (constant vs non-constant), network type (association vs homogeneous), and asocial parameters (aviary effect vs no aviary effect). Support for same *s* versus different *s* was calculated as the sum of the weights of the 48 models containing same *s* versus the sum of the weights of the 48 models containing different *s*. Similarly, network type consisted of 32 models for each different possible combination of network (all, adults only, juveniles only).

SUPPLEMENTAL REFERENCES

S1. Psorakis, I., Roberts, S.J., Rezek, I., and Sheldon, B.C. (2012). Inferring social network structure in ecological systems from spatio-temporal data streams. J R Soc Interface *9*, 3055-3066.

S2. Psorakis, I., Voelkl, B., Garroway, C.J., Radersma, R., Aplin, L.M., Crates, R.A., Culina, A., Farine, D.R., Firth, J.A., Hinde, C.A., et al. (2015). Inferring social structure from temporal data. Behav Ecol Sociobiol *69*, 857-866.

S3. Farine, D.R. (2013). Animal Social Network Inference and Permutations for Ecologists in R using asnipe. Methods Ecol Evol *4*, 1187–1194.

S4. Franz, M., and Nunn, C.L. (2009). Network-based diffusion analysis: a new method for detecting social learning. P Roy Soc B-Biol Sci *276*, 1829-1836.

S5. Hoppitt, W., and Laland, K.N. (2011). Detecting Social Learning Using Networks: A Users Guide. Am J Primatol *73*, 834-844.

S6. Hoppitt, W., Boogert, N.J., and Laland, K.N. (2010). Detecting social transmission in networks. J Theor Biol *263*, 544-555.

S7. Farine, D.R., Aplin, L.M., Sheldon, B.C., and Hoppitt, W. (2015). Interspecific social networks promote information transmission in wild songbirds. P Roy Soc B-Biol Sci *282*, 20142804.
