# Supplementary material for: Early-Life Stress Triggers Juvenile Zebra Finches to Switch Social Learning Strategies
Source: Curr Biol. 2015 Aug 17;25(16):2184–8. doi: 10.1016/j.cub.2015.06.071 (PMC4540255; doi:10.1016/j.cub.2015.06.071)
Supplement: Document S2. Article plus Supplemental Information [file mmc2.pdf]

Report

Current Biology

Early-Life Stress Triggers Juvenile Zebra Finches to Switch Social Learning Strategies

Highlights

- Juvenile zebra finches learn foraging skills from their parents
- Stress hormone exposure triggers juveniles to learn from unrelated adults instead
- Stress may be a cue juveniles use to inform their behavioral strategies
- Switching social learning strategy may alter developmental trajectories adaptively

Authors

Damien R. Farine, Karen A. Spencer, Neeltje J. Boogert

Correspondence

damien.farine@zoo.ox.ac.uk

In Brief

Farine et al. demonstrate that young birds learn foraging skills from their parents. However, early-life exposure to experimentally elevated stress hormones prompts juveniles to switch strategies and learn only from unrelated adults. This may help them to compensate for their poor natal environment and acquire more adaptive behaviors in nature.

CrossMark

Farine et al., 2015, Current Biology *25*, 2184–2188
August 17, 2015 ©2015 The Authors
http://dx.doi.org/10.1016/j.cub.2015.06.071

CellPress

CellPress
Current Biology
Report

Early-Life Stress Triggers Juvenile Zebra Finches to Switch Social Learning Strategies

Damien R. Farine,[1,2,3,*] Karen A. Spencer,[4] and Neeltje J. Boogert[4,5]
[1]Edward Grey Institute of Field Ornithology, Department of Zoology, University of Oxford, Oxford OX1 3PS, UK
[2]Department of Anthropology, University of California, Davis, Davis, CA 95616, USA
[3]Smithsonian Tropical Research Institute, Panamá 0843-03092, Panama
[4]School of Psychology and Neuroscience, University of St. Andrews, St. Andrews KY16 9JP, UK
[5]Department of Zoology, University of Cambridge, Cambridge CB2 3EJ, UK
*Correspondence: damien.farine@zoo.ox.ac.uk
http://dx.doi.org/10.1016/j.cub.2015.06.071
This is an open access article under the CC BY license (http://creativecommons.org/licenses/by/4.0/).

SUMMARY

Stress during early life can cause disease and cognitive impairment in humans and non-humans alike [1]. However, stress and other environmental factors can also program developmental pathways [2, 3]. We investigate whether differential exposure to developmental stress can drive divergent social learning strategies [4, 5] between siblings. In many species, juveniles acquire essential foraging skills by copying others: they can copy peers (horizontal social learning), learn from their parents (vertical social learning), or learn from other adults (oblique social learning) [6]. However, whether juveniles' learning strategies are condition dependent largely remains a mystery. We found that juvenile zebra finches living in flocks socially learned novel foraging skills exclusively from adults. By experimentally manipulating developmental stress, we further show that social learning targets are phenotypically plastic. While control juveniles learned foraging skills from their parents, their siblings, exposed as nestlings to experimentally elevated stress hormone levels, learned exclusively from unrelated adults. Thus, early-life conditions triggered individuals to switch strategies from vertical to oblique social learning. This switch could arise from stress-induced differences in developmental rate, cognitive and physical state, or the use of stress as an environmental cue. Acquisition of alternative social learning strategies may impact juveniles' fit to their environment and ultimately change their developmental trajectories.

RESULTS AND DISCUSSION

Social learning, where animals learn from observing or interacting with others, enables traditions to be transmitted across generations [4]. Social structure can greatly affect information spread [7–9] and the transmission of novel behaviors [10–13], while individuals' position within their social network can alter their fitness [14–16]. However, it is unclear whether individuals' characteristics modulate information transmission through social networks: do individuals pay equal attention to all their associates? If not, what strategies do they use to decide who to learn from [5], and how are these influenced by the environment, both past and present?

One major determinant of individual variation in social behavior, and potentially social learning, is exposure to stress in early life [17]. Developmental stress has been linked to variation in dispersal distance [18], patterns of social contacts [17], and information use [19]. We hypothesize that developmental stress could also guide social learning strategies, in terms of who to copy when faced with novel environmental challenges. Here we investigate whether (1) individuals of the highly gregarious zebra finch (*Taeniopygia guttata*) are biased in whom they learn from and (2) juveniles exposed to experimentally elevated stress hormone levels in early life later adjust their learning strategies. Zebra finches use social learning to acquire their songs and song preferences [20], when choosing mates [21], and when deciding where and what to eat [22]. Here we focus on the social acquisition of foraging skills.

To determine how developmental stress affects social learning strategies, we exposed half of the chicks in each of 13 broods to physiologically relevant doses of the avian stress hormone corticosterone (CORT) on days 12–28 post-hatching. Once chicks reached nutritional independence at ∼35 days, we released six to seven families into each of two identical aviaries (N = 29 and 34 finches, respectively). This resembles flock composition in the wild, where neighboring families forage together for food (unpublished data). For 20 days, we collected a complete record of all birds' foraging associations from passive integrated transponder (PIT) tags fitted to each bird and detected by radio-frequency identification (RFID) antennae fitted to two feeders in each aviary. We then introduced a novel foraging task [23, 24] on day 21 and measured each individual's latency to first approach and to first solve (see the Experimental Procedures for details and Table S1 for descriptive statistics). Of the 63 birds, 39 solved the task. These solvers represented 11 of the 26 adults and 28 of the 37 juveniles. Half of the 28 juvenile solvers were controls, and half were treated with CORT.

Individuals Copy Adults to Acquire Novel Foraging Skills

We quantified social information transmission in each aviary by combining the 20-day social foraging network with the birds' task-solving latencies in a network-based diffusion analysis

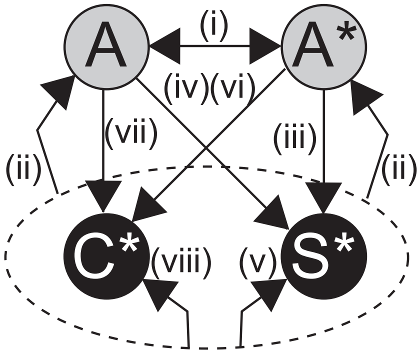

Figure 1. Summary of Edge Classifications

The full network was partitioned into eight different networks, each containing a different class of edge (see the main text), and different combinations of these were used in an information-theoretic framework to evaluate our hypotheses (see also Figure S1). Gray nodes (A) are adults; black nodes are juveniles, split into control (C) and CORT/developmentally stressed (S) treatments. The * represents individuals from the same family (thus, here one adult is a parent and the other is unrelated). Edges from all juveniles (dashed oval) represent edges from related and unrelated juveniles combined (both C and S treatments and all unrelated juveniles are included).

(NBDA) [25, 26]. NBDA quantifies the rates at which individuals acquire information or novel traits following a previously measured social network. It estimates how much individuals' social learning rates are accelerated, or the likelihood of learning a novel task solution increased, when their associates demonstrate this new information (represented by the parameter *s*; see the Supplemental Experimental Procedures). NBDA has generated significant insights into how animals acquire social information about novel food locations and foraging behaviors [7, 9–12, 27]. NBDA of a single task solution cannot distinguish between imitation and socially facilitated learning (i.e., demonstrators attract naive associates to the task, where the latter then learn asocially). However, here we are interested in social learning strategies in terms of who learns (either directly or indirectly) from whom, rather than in the social learning mechanism involved.

We used a recently developed variant [9] of NBDA that quantifies transmission rates between different types of social network connections ("edges"). Instead of estimating a single social transmission rate between all types of individuals (regular NBDA), we partitioned the edges into eight separate directed networks (Figure 1) containing all the incoming edges from adults to adults (i), juveniles to their parents and unrelated adults (ii), parents to their CORT-treated offspring (iii), adults to unrelated CORT-treated juveniles (iv), control and CORT juveniles to CORT juveniles (v), parents to their control offspring (vi), adults to unrelated control juveniles (vii), and control and CORT juveniles to control juveniles (viii). The sum of these eight networks is the observed network (i.e., no edges occurred in more than one network). For each network, we estimated a separate rate of social transmission *s* for each category of connections. For example, if juveniles learned exclusively from each other, then we would expect a high *s* for the juvenile to juvenile network and *s* = 0 for other networks.

We used an information-theoretic approach, constructing all possible models and comparing these to evaluate our hypotheses (see the Supplemental Experimental Procedures and Figure S1). We used corrected Akaike's information criterion to allow for model uncertainty, summing up the Akaike weights to calculate the level of support for each hypothesis (following [28]). Analysis of the data from both diffusions (one in each aviary) revealed that information spread through the foraging association networks (supported by 99.7% of model weights; Table 1) rather than through homogeneous networks (i.e., where all associations are set to 1; supported by 0.3% of model weights), or via asocial learning. There was also evidence (10.7% of model weights) for a 2.5% faster learning rate in the second aviary. Table S2 contains the top five models, accounting for >98% of model weights.

Table 1. Relative Importance of Three Major Pathways of Information Transfer

Network	All Conspecifics	Adults Only	Juveniles Only	Total
Association	17.7	81.7	0.3	99.7
Homogeneous	<0.01	<0.01	0.3	0.3
Total	17.7	81.7	0.6	

Summary of the total Akaike weight (%) for all models testing the hypotheses that individuals learned the novel foraging task solution from all classes of conspecifics, individuals learned it exclusively from adults, and individuals learned it exclusively from juveniles. Models were all additive (see Table S4 for weights from multiplicative models). Networks therein were either foraging association informed or homogeneous (see the Supplemental Experimental Procedures). Support for asocial models was 3.69×10^{-21}.

We estimated the relative importance of three major pathways of information transmission: (1) individuals learned from everyone, (2) individuals learned exclusively from adults, and (3) individuals learned exclusively from juveniles (Figure S1). Models containing transmission from adults only were best supported (total Akaike weight = 81.7%; Table 1), suggesting that both adults and juveniles learned almost exclusively from adults. These results provide some of the strongest empirical support yet for "directed social learning" [29] in a naturalistic, family-structured social context. This is consistent with the notion that individuals should tailor their strategies to acquire relevant traits. Similarly, primates tend to copy higher-ranking, i.e., nominally more successful, conspecifics [30–32].

Developmental Stress Modulates Juveniles' Social Learning Strategies

We then tested whether social learning rates were the same across each network (same *s*) or differed in each network (different *s*; see Figure S1). We found strong evidence for a different *s* for each network (total Akaike weight = 99.3%; Table 2) and for differences in social learning strategies among juveniles. Using the Akaike weights for each model, we obtained the model-averaged estimates for each *s* (Table 3). Rates of transmission (*s*) differed between control and CORT-treated juveniles. Control juveniles relied more on their parents (*s* = 9.9) than on unrelated adults (*s* = 6.6) to learn the novel foraging skill. That is, one unit of social network connection to a knowledgeable parent increased control juveniles' likelihood of learning the behavior by one-third compared to a unit of social network

Table 2. Relative Support for Uniform versus Varying Rates of Transmission across Different Networks

Network	Same *s*	Different *s*	Total
Association	0.4	99.3	99.7
Homogeneous	0.3	0.04	0.3
Total	0.7	99.3	

Summary of the total Akaike weight (%) for all models testing the hypotheses that *s* was the same across all networks in each model or *s* differed across all networks in each model. Models were all additive (see Table S5 for weights from multiplicative models). Networks therein were either foraging association informed or homogeneous (see the Supplemental Experimental Procedures). Support for asocial models was 3.69×10^{-21}.

Table 3. Model-Averaged Estimates of Information Transmission Rates between Classes of Individual

Network	Edges From	Edges To	Social Learning Rate (*s*)	Upper 95% CI	Lower 95% CI
i	adults	adults	2.22	5.08	0.32
ii	CORT and control juveniles	adults	0.006	0.07	0
iii	parents	CORT-treated juveniles	0.005	0.08	0
iv	unrelated adults	CORT-treated juveniles	5.75	10.88	2.29
v	CORT and control juveniles	CORT-treated juveniles	0.004	0.10	0
vi	parents	control juveniles	9.86	18.26	6.29
vii	unrelated adults	control juveniles	6.62	11.71	2.08
viii	CORT and control juveniles	control juveniles	0.13	0.25	0

Each network contained the directed social network links from individuals of a given class (e.g., parents) to another class (e.g., offspring). This approach provides social learning rate estimates per unit of social network connection to knowledgeable individuals for each class independently (given by *s* in the models; see the Supplemental Experimental Procedures). CI, confidence interval. See also Table S3.

connection to a knowledgeable but unrelated adult. In contrast, the model-averaged rate of information transmission from parents to CORT-treated juveniles was very low ($s = 0.005$). This is despite CORT-treated and control juveniles having similar foraging association strengths to parents (mean = 0.31 and 0.32, respectively). Instead, CORT-treated juveniles learned almost exclusively from unrelated adults ($s = 5.7$). Relative transmission rates were similar in the two aviaries (Table S3). These results suggest that increased exposure to stress hormones during post-natal development resulted in a switch by juveniles from vertical to oblique social learning strategies. The extent to which this switch was driven by active model choice by juveniles, rather than a parental decision to be more tolerant toward their control offspring, is an interesting question for future research, although we never observed adult aggression toward juveniles.

Social Learning Rates Are State Dependent

Naive adults and juveniles varied in their latencies to solve the task and their reliance on social information. Naive adults were slower than juveniles at *approaching* the task (linear mixed-effects model [LMM] of task approach latencies [all models herein include "family" nested within "aviary" as random effects]: estimate ± SE = 3684.31 ± 1662.66, $t_{41} = 2.22$, p = 0.032), but *solved* the task faster (LMM of solve latency: estimate ± SE = −7370.60 ± 3115.71, $t_{25} = -2.37$, p = 0.026). However, once the task was being demonstrated, every unit of social network connection to a knowledgeable adult increased adults' learning rate by only 2.5 times. Naive juveniles were at least twice as likely as naive adults to acquire the behavior from adult demonstrators: their social learning rate increased by at least 5.7 times the baseline rate per unit increase in network connection to knowledgeable adults. Higher rates of social learning in juveniles as compared to adults have also been reported in blue tits (*Cyanistes caeruleus*) [33], great tits (*Parus major*) [11], and white-throated magpies (*Calocitta formosa*) [34].

Because information about the novel foraging task was transmitted socially, who learned was largely dependent on which adults demonstrated the task and the propensity of juveniles to forage with these demonstrators. Control and CORT-treated juveniles learned from unrelated adults at similar rates (Table 3), suggesting that they did not differ in their *ability* to acquire the trait socially when using the same category of demonstrators. However, CORT-treated juveniles started to solve the task sooner than control juveniles (LMM of solve latency: estimate ± SE = −7603.62 ± 3250.04, $t_{14} = -2.34$, p = 0.035). Thus, even though control juveniles learned rapidly from their parents, whereas CORT-treated juveniles did not, CORT-treated juveniles still acquired the trait sooner. This could be because they relied more on individual trial-and-error learning [35]. Alternatively, CORT-treated juveniles may have simply had access to information about the task sooner by associating with more unrelated adults (mean network association strength of 1.62 versus 1.57 for control juveniles), who made up the majority of (potentially demonstrating) adults in each aviary.

Conclusions

The social network guided the transmission of a novel foraging task solution through flocks of birds, but not all connections had an equal likelihood of transmitting information. Importantly, despite both relying on social learning from adults when acquiring the novel foraging skill, CORT-treated and control juveniles differed in their social learning strategies. Control juveniles largely copied their parents to acquire the novel foraging skill. CORT-treated juveniles, in contrast, relied on learning from unrelated adults only.

Developmental stress may induce switches in social learning strategies in various ways. These may involve changes in developmental rate [36], stress responsiveness [36], or cognitive and social skills [1]. However, these cannot completely explain why CORT-treated juveniles did not acquire the novel foraging task solution from their parents, despite associating with them almost as strongly as did control juveniles in the social foraging network.

Theory suggests that developmental stress may be used as an informative cue about an individual's environment [2, 37], which could range from parental investment to natal habitat quality. If so, it may enable juveniles to avoid becoming trapped in a negative feedback loop provided by a bad start in life, by programming them to adopt alternative, and potentially more adaptive, behaviors.

EXPERIMENTAL PROCEDURES

Rearing and Hormone Treatment

We individually housed 13 domesticated zebra finch pairs and synchronized the within-brood hatching dates of their eggs by replacing them with plastic dummies until the brood was complete. Chicks were individually marked, and approximately half in each brood were randomly assigned to the following experimental CORT treatment [19]: they were fed 20 μl of CORT (Sigma Aldrich; 0.155 mg/ml in peanut oil) twice daily, giving a total dose of 6.2 μg CORT/day. This dose is known to result in plasma CORT levels comparable to those naturally induced in untreated chicks exposed to an acute stressor [36]. Control chicks were fed 20 μl of pure peanut oil when their siblings received CORT. Experiments were conducted under Home Office Animals (Scientific) Procedures Act project license no. 60/4068 and personal license no. 60/13491.

Free-Flying Aviaries

When chicks were 37 ± 1 days old, we fitted them and their parents with PIT tags attached to unique color rings and released families together into one of two identical aviaries (3 × 3.1 × 3.2 m) on the same day. Aviaries contained seven (N = 34 birds) and six families (N = 29 birds), respectively, and both were equipped with two identical transparent feeders containing finch seed at all times, except during the novel foraging task experiment (see below). Feeders were fitted with RFID antennae to record the PIT tags of zebra finches as they freely entered and exited the feeders.

Inferring the Social Network

The data loggers attached to the RFID antennae provided a complete record of individuals visiting the feeders simultaneously. From this temporal data stream, we extracted bouts of foraging activity using a well-established algorithm [38, 39] and used the simple ratio index to calculate association strengths (see the Supplemental Experimental Procedures) with the asnipe package [40] in R.

Novel Foraging Tasks

On the mornings of days 21–23, we removed feeders at 9:00 a.m. and, after 1 hr of food deprivation, presented a novel foraging task on a platform (1 × 1 × 1 m) in each aviary. This task consisted of four white plastic foraging grids (8 × 12 × 2 cm), each containing 12 wells (2-cm diameter, 1.5-cm depth; 48 wells in total). Each well contained spinach (0.5 × 0.5 cm) covered with a lid. Lids consisted of yellow cardboard squares (2 × 2 cm) with upward-folded corners and felt bumpers (2-cm diameter, 0.5-cm height). The same baited grids had been presented for 2 days preceding the experiment, and four lids were added on top of each grid (but not covering the wells) 1 day before the experiment. This habituated birds to the novel objects and prevented neophobia from inhibiting skill acquisition.

The zebra finches were left to discover how to remove the lids from the wells to obtain the food reward, which we filmed from different angles. We returned to the aviaries each hour to re-bait the grids, for a total of three 1-hr trials per day over 3 days. At the end of each test day, foraging grids were removed from the aviary and the regular feeders were returned. From the videos, we scored the latency (counted in seconds from the start of the experiment, excluding times when the task was being re-baited/not presented) of each bird's first approach within pecking distance of a lid, as well as their first and all subsequent task solutions. A bird was considered to have solved the task when it deliberately lifted the lid completely out of the well (i.e., not accidentally kicking/knocking it off) so that it could access the spinach underneath. We identified individuals from the videos using their unique color rings. We considered the latency of each bird's first task solve to be the time point at which it switched from a naive to an informed state. Table S1 contains task performance statistics. We then used these latencies to model the spread of this information through the zebra finch flocks using NBDA. Full details and model specifications are provided in the Supplemental Experimental Procedures.

SUPPLEMENTAL INFORMATION

Supplemental Information includes Supplemental Experimental Procedures, one figure, and five tables and can be found with this article online at http://dx.doi.org/10.1016/j.cub.2015.06.071.

AUTHOR CONTRIBUTIONS

N.J.B. and K.A.S. devised the experiment, N.J.B. collected the data, D.R.F. performed the analysis, D.R.F. and N.J.B. drafted the manuscript, and all authors contributed to revisions. D.R.F. and N.J.B. are equal contributors.

ACKNOWLEDGMENTS

We thank Ben Sheldon, Kevin Laland, Will Hoppitt, Lucy Aplin, Bram Kuijper, and Willem Frankenhuis for their constructive feedback, James Sturdy for his help scoring the videos, and Roland Stump for his help setting up the PIT/RFID system. D.R.F. was funded by grants from the NSF (NSF-IOS1250895) to Margaret Crofoot and BBSRC (BB/L006081/1) to Ben Sheldon, K.A.S. was funded by a BBSRC David Phillips Research Fellowship, and N.J.B. was funded by a Netherlands Organization for Scientific Research (NWO) Rubicon grant.

Received: March 9, 2015
Revised: May 27, 2015
Accepted: June 29, 2015
Published: July 23, 2015

REFERENCES

1. Lupien, S.J., McEwen, B.S., Gunnar, M.R., and Heim, C. (2009). Effects of stress throughout the lifespan on the brain, behaviour and cognition. Nat. Rev. Neurosci. *10*, 434–445.
2. Leimar, O., Hammerstein, P., and Van Dooren, T.J.M. (2006). A new perspective on developmental plasticity and the principles of adaptive morph determination. Am. Nat. *167*, 367–376.
3. Sih, A. (2011). Effects of early stress on behavioral syndromes: an integrated adaptive perspective. Neurosci. Biobehav. Rev. *35*, 1452–1465.
4. Hoppitt, W., and Laland, K.N. (2013). Social Learning: An Introduction to Mechanisms, Methods and Models (Princeton: Princeton University Press).
5. Laland, K.N. (2004). Social learning strategies. Learn. Behav. *32*, 4–14.
6. McElreath, R., and Strimling, P. (2008). When natural selection favors imitation of parents. Curr. Anthropol. *49*, 307–316.
7. Aplin, L.M., Farine, D.R., Morand-Ferron, J., and Sheldon, B.C. (2012). Social networks predict patch discovery in a wild population of songbirds. Proc. Biol. Sci. *279*, 4199–4205.
8. Atton, N., Galef, B.J., Hoppitt, W., Webster, M.M., and Laland, K.N. (2014). Familiarity affects social network structure and discovery of prey patch locations in foraging stickleback shoals. Proc. Biol. Sci. *281*, 20140579.
9. Farine, D.R., Aplin, L.M., Sheldon, B.C., and Hoppitt, W. (2015). Interspecific social networks promote information transmission in wild songbirds. Proc. Biol. Sci. *282*, 20142804.
10. Allen, J., Weinrich, M., Hoppitt, W., and Rendell, L. (2013). Network-based diffusion analysis reveals cultural transmission of lobtail feeding in humpback whales. Science *340*, 485–488.
11. Aplin, L.M., Farine, D.R., Morand-Ferron, J., Cockburn, A., Thornton, A., and Sheldon, B.C. (2015). Experimentally induced innovations lead to persistent culture via conformity in wild birds. Nature *518*, 538–541.

12. Boogert, N.J., Nightingale, G.F., Hoppitt, W., and Laland, K.N. (2014). Perching but not foraging networks predict the spread of novel foraging skills in starlings. Behav. Processes *109* (Pt B), 135–144.
13. Claidière, N., Messer, E.J.E., Hoppitt, W., and Whiten, A. (2013). Diffusion dynamics of socially learned foraging techniques in squirrel monkeys. Curr. Biol. *23*, 1251–1255.
14. Farine, D.R., and Sheldon, B.C. (2015). Selection for territory acquisition is modulated by social network structure in a wild songbird. J. Evol. Biol. *28*, 547–556.
15. McDonald, D.B. (2007). Predicting fate from early connectivity in a social network. Proc. Natl. Acad. Sci. USA *104*, 10910–10914.
16. Silk, J.B., Alberts, S.C., and Altmann, J. (2003). Social bonds of female baboons enhance infant survival. Science *302*, 1231–1234.
17. Boogert, N.J., Farine, D.R., and Spencer, K.A. (2014). Developmental stress predicts social network position. Biol. Lett. *10*, 20140561.
18. Tschirren, B., Fitze, P.S., and Richner, H. (2007). Maternal modulation of natal dispersal in a passerine bird: an adaptive strategy to cope with parasitism? Am. Nat. *169*, 87–93.
19. Boogert, N.J., Zimmer, C., and Spencer, K.A. (2013). Pre- and post-natal stress have opposing effects on social information use. Biol. Lett. *9*, 20121088.
20. Slater, P.J., Eales, L.A., and Clayton, N. (1988). Song learning in zebra finches (Taeniopygia guttata): progress and prospects. Adv. Study Behav. *18*, 1–34.
21. Swaddle, J.P., Cathey, M.G., Correll, M., and Hodkinson, B.P. (2005). Socially transmitted mate preferences in a monogamous bird: a non-genetic mechanism of sexual selection. Proc. Biol. Sci. *272*, 1053–1058.
22. Riebel, K., Spierings, M.J., Holveck, M.J., and Verhulst, S. (2012). Phenotypic plasticity of avian social-learning strategies. Anim. Behav. *84*, 1533–1539.
23. Boogert, N.J., Reader, S.M., Hoppitt, W., and Laland, K.N. (2008). The origin and spread of innovations in starlings. Anim. Behav. *75*, 1509–1518.
24. Templeton, C.N., Laland, K.N., and Boogert, N.J. (2014). Does song complexity correlate with problem-solving performance in flocks of zebra finches? Anim. Behav. *92*, 63–71.
25. Franz, M., and Nunn, C.L. (2009). Network-based diffusion analysis: a new method for detecting social learning. Proc. Biol. Sci. *276*, 1829–1836.
26. Hoppitt, W., and Laland, K.N. (2011). Detecting social learning using networks: a users guide. Am. J. Primatol. *73*, 834–844.
27. Hobaiter, C., Poisot, T., Zuberbühler, K., Hoppitt, W., and Gruber, T. (2014). Social network analysis shows direct evidence for social transmission of tool use in wild chimpanzees. PLoS Biol. *12*, e1001960.
28. Burnham, K.P., and Anderson, D.R. (2002). Model selection and Multimodel Inference: A Practical Information-Theoretic Approach (New York: Springer-Verlag).
29. Coussi-Korbel, S., and Fragaszy, D.M. (1995). On the relation between social dynamics and social learning. Anim. Behav. *50*, 1441–1453.
30. Horner, V., Proctor, D., Bonnie, K.E., Whiten, A., and de Waal, F.B.M. (2010). Prestige affects cultural learning in chimpanzees. PLoS ONE *5*, e10625.
31. van de Waal, E., Borgeaud, C., and Whiten, A. (2013). Potent social learning and conformity shape a wild primate’s foraging decisions. Science *340*, 483–485.
32. van de Waal, E., Renevey, N., Favre, C.M., and Bshary, R. (2010). Selective attention to philopatric models causes directed social learning in wild vervet monkeys. Proc. Biol. Sci. *277*, 2105–2111.
33. Aplin, L.M., Sheldon, B.C., and Morand-Ferron, J. (2013). Milk bottles revisited: social learning and individual variation in the blue tit, Cyanistes caeruleus. Anim. Behav. *85*, 1225–1232.
34. Langen, T.A. (1996). Social learning of a novel foraging skill by white-throated magpie-jays (Calocitta formosa, Corvidae): a field experiment. Ethology *102*, 157–166.
35. Crino, O.L., Driscoll, S.C., Ton, R., and Breuner, C.W. (2014). Corticosterone exposure during development improves performance on a novel foraging task in zebra finches. Anim. Behav. *91*, 27–32.
36. Spencer, K.A., Evans, N.P., and Monaghan, P. (2009). Postnatal stress in birds: a novel model of glucocorticoid programming of the hypothalamic-pituitary-adrenal axis. Endocrinology *150*, 1931–1934.
37. English, S., Pen, I., Shea, N., and Uller, T. (2015). The information value of non-genetic inheritance in plants and animals. PLoS ONE *10*, e0116996.
38. Psorakis, I., Roberts, S.J., Rezek, I., and Sheldon, B.C. (2012). Inferring social network structure in ecological systems from spatio-temporal data streams. J. R. Soc. Interface *9*, 3055–3066.
39. Psorakis, I., Voelkl, B., Garroway, C.J., Radersma, R., Aplin, L.M., Crates, R.A., Culina, A., Farine, D.R., Firth, J.A., Hinde, C.A., et al. (2015). Inferring social structure from temporal data. Behav. Ecol. Sociobiol. *69*, 857–866.
40. Farine, D.R. (2013). Animal social network inference and permutations for ecologists in R using asnipe. Methods Ecol. Evol. *4*, 1187–1194.

Current Biology

Supplemental Information

Early-Life Stress Triggers Juvenile Zebra Finches to Switch Social Learning Strategies

Damien R. Farine, Karen A. Spencer, and Neeltje J. Boogert

Figure S1: Framework used to partition the different categories of edges in the observed foraging network to evaluate two hypotheses: 1) the major pathways of information transmission: (a) individuals learnt from everyone (all eight networks were included, i.e. the sum of these networks equals the foraging network), (b) individuals learnt exclusively from adults, where *s* for networks with edges originating from juveniles (i.e. networks (ii), (v) and (viii)) was constrained to 0, or (c) individuals learnt exclusively from juveniles, where *s* for networks with edges originating from adults (i.e. networks (i), (iii), (iv), (vi), (vii)), was constrained to 0, and 2) whether *s* was the same or different in each network. Networks are described in Figure 1. Here, A (red nodes) are adults, and juveniles (blue nodes inside dashed oval) are partitioned into control chicks (C) and stressed (CORT) chicks (S). Individuals marked with a * are members of the same family (thus, one adult is a parent and the other is unrelated in this diagram).

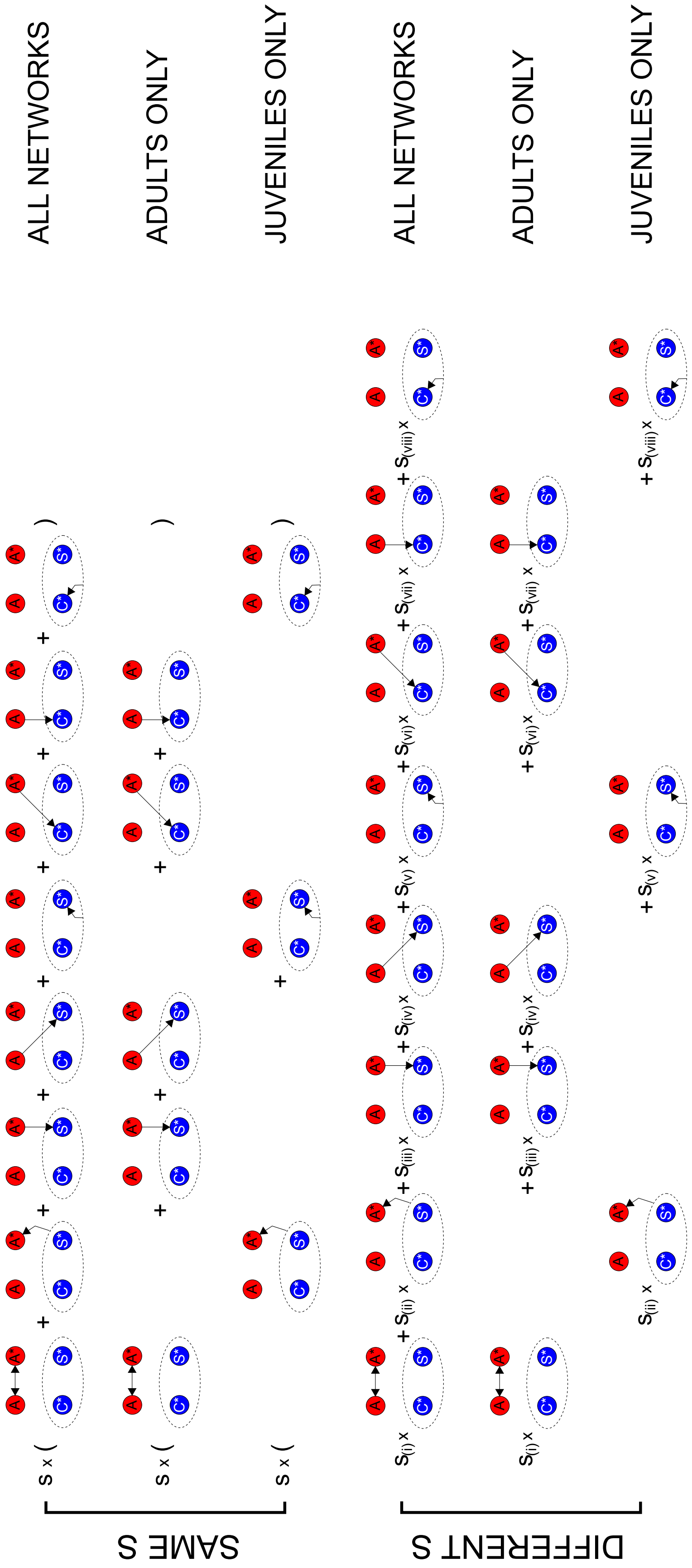

Table S1: Descriptive statistics for approach and task solving latencies in seconds across the two aviaries

	Latency to approach task			Latency to solve task		
	**Adults (*n*=19)**	**Control juveniles (*n*=16)**	**CORT juveniles (*n*=20)**	**Adults (*n*=11)**	**Control juveniles (*n*=14)**	**CORT juveniles (*n*=14)**
n in aviary 1	9	8	11	7	8	8
n in aviary 2	10	8	9	4	6	6
Mean	11328	7758	6743	8534	20205	12602
Median	7837	7464	4624	7828	21107	9807
Range	28814	15069	28363	28827	31694	27571
Min	2327	2509	2352	2339	2584	2566
Max	31141	17578	30715	31166	34278	30137

Table S2: Top 5 models ranked by Akaike weight. The best-supported models had a mixture of constant and non-constant asocial learning rates (i.e. decreasing over time, but the rate of decrease was small at only 0.75), all were additive, and all included the foraging-association informed rather than a homogeneous social network. Subsequent models had an Akaike weight < 1%.

**Model rank**	**Incoming edges from**	***s* different or same across networks**	**Baseline**	**Aviary difference**	**df**	**AICc**	**Akaike weight (%)**
1	Adults only	Different	Non-constant	NA	8	898.48	54.2
2	All	Different	Constant	NA	10	900.78	17.2
3	Adults only	Different	Constant	NA	7	900.79	17.1
4	Adults only	Different	Non-constant	0.62	9	901.99	9.4
5	Adults only	Different	Constant	2.64	8	906.80	0.8

Table S3: Estimates of information transmission rates (*s*) between classes of individual using the top model (Table S2) and run for each aviary separately. Each network fit an *s* parameter to the social network links from individuals of a given class (e.g. parents) to another class (e.g. offspring). The top model constrained *s* parameters for edges from juveniles (marked by a *) to 0 (i.e. the model was an 'adults only' model).

**Network**	**Edges from:**	**Edges to:**	**Aviary 1**	**Aviary 2**
i	Adults	Adults	1.6	2.8
ii	CORT & control juveniles	Adults	0*	0*
iii	Parents	CORT-treated juveniles	0	0
iv	Unrelated adults	CORT-treated juveniles	3.1	6.7
v	CORT & control juveniles	CORT-treated juveniles	0*	0*
vi	Parents	Control juveniles	9.7	12.1
vii	Unrelated adults	Control juveniles	2.95	8.6
viii	CORT & control juveniles	Control juveniles	0*	0*

Table S4: Summary of the total Akaike weight (%) for all *multiplicative* models testing the hypotheses that (i) individuals learnt the novel foraging task solution from all classes of conspecifics, (ii) individuals learnt it exclusively from adults, and (iii) individuals learnt it exclusively from juveniles. Networks therein were either foraging association-informed or homogeneous (see Supplemental Experimental Procedures). Support for asocial models was 3.69x10-21.

**Network**	**(i) All conspecifics**	**(ii) Adults only**	**(iii) Juveniles only**	***Total***
Association	6.3x10-8	1.6x10-7	1.6x10-5	*7.1x10-5*
Homogeneous	8.0x10-8	5.3x10-8	5.3x10-6	*7.4x10-5*
**Total**	1.4x10-7	**2.1x10-7**	**2.1x10-5**	

Table S5: Summary of the total Akaike weight (%) for all *multiplicative* models testing the hypotheses that (i) *s* is the same across all networks in each model, or (ii) *s* differs across all networks in each model. Networks therein were either foraging association-informed or homogeneous (see Supplemental Experimental Procedures). Support for asocial models was 3.69x10-21.

**Network**	**(i) *S* same**	**(ii) *S* different**	***Total***
Association	4.7x10-5	2.4x10-5	*7.1x10-5*
Homogeneous	5.8x10-5	1.7x10-5	*7.4x10-5*
**Total**	**1.0x10-4**	**4.1x10-5**	

SUPPLEMENTAL EXPERIMENTAL PROCEDURES

Inferring the social network

We constructed the social network using the following method: (i) individuals were individually fitted with unique (10-digit hexadecimal code) electronic Passive Integrated Transponder (PIT) tags attached to a plastic, uniquely coloured, leg ring. (ii) Tags were recorded by RFID antennae integrated into the feeder entrances and the bird identity (PIT tag code), feeder identity, time and date of each feeder visit were saved onto the internal memory of the PIT-tag reader devices (Dorset ID, The Netherlands). (iii) Foraging groups were identified by extracting peaks of feeder visit activity in the temporal data stream using a clustering algorithm (i.e. a Gaussian-mixed model [S1, S2]). On average, such foraging groups spent 290 seconds at the feeder, and contained 7 individuals. (iv) The association strength (edge weights in the network) between each dyad of birds in each aviary was defined as the rate of feeding co-occurrence, calculated as the number of observations of both individuals in the same foraging group divided by the number of observations of at least one individual in a foraging group (the simple ratio index which ranges from 0: never observed together, to 1: always observed together). While observing two individuals in the same foraging group may not necessarily be meaningful for a single event, this approach provides highly robust estimates of foraging associations through repeated observations of dyads, in this case 4216 group observations in aviary 1 and 4077 group observations in aviary 2. This also represents a complete record of every single visit to each feeder by all individuals in each aviary over the 20 observation days. (v) We created a single network combining the data from all 20 days of observation, calculated using the asnipe library [S3] in R.

Network-based diffusion analysis (NBDA)

In the standard NBDA model [S4-S6], the rate at which an individual adopts a new trait is estimated as a function of its connections to knowledgeable individuals (“demonstrators”). The effect of these demonstrators is scaled by the parameter *s*, which is the rate of transmission per unit of social network connection to demonstrators. The multi-NBDA model builds on this by including multiple candidate networks, each of which can have a different value for *s* (cf different rates of transmission through different networks) [S7]. We partitioned the zebra finch foraging association network into eight networks (given in the main text, see also Table 3), each representing a sub-network of the complete foraging network from each aviary and containing a single class of edges (see Figure S1). Each network contained the full set of individuals, where those that were not connected to any edges of the given class (for example adults in a juvenile-to-juvenile network) were isolates (i.e. not connected to anyone). We also tested asocial learning models (i.e. no edges in the network) and models of undifferentiated or “homogeneous” social transfer (i.e. fully-connected network where every edge = 1). We used the continuous variant of multi-NBDA, and included either a constant or varying (i.e. becoming faster or slower) baseline rate of transmission over time. We combined the data from the two aviaries when estimating parameter values (using each aviary’s network with its diffusion data), and included “aviary” as an asocial parameter to account for potential differences in transmission rates between them. We used model-averaging to evaluate different hypotheses and estimate transmission rates through each network. The parameters for each model were fitted using the optim function in R.

Equations

We used a recently-developed variant of network-based diffusion analysis (NBDA) that allows transmission rates to be quantified across multiple candidate networks [S7]. The functional form of this model for M networks is given by:

$$\lambda_i(t) = \lambda_0(t)\left(\sum_{k=1}^{M}\left[s_k \sum_{j=1}^{N} a_{ijk} z_j(t)\right] + 1\right)\left(1 - z_i(t)\right)$$

where $\lambda_0(t)$ is the baseline rate of acquisition (herein referred to as asocial learning when $s = 0$), s_k is the rate of social transmission through the edges given by network k, a_{ijk} is the edge weight between individuals i and j in network k (all networks had the same N individuals, but all $a_{ij} = 0$ for nodes that were not connected to edges represented in network k, such as adults in networks v and viii (see Table 3)), and $z_i(t)$ is the status of individual i at time t (0 = naive, 1 = informed). Because diffusions occurred in different aviaries, we also included an asocial variable $LP_i = \beta x_i$, where x_i is an indicator variable showing whether individual i was in aviary 2 ($x_i = 1$) or aviary 1 ($x_i = 0$), to account for potential differences in baseline rates of learning between the two aviaries. If models containing this asocial variable are better supported than models not containing it (see below), then it suggests that individuals' learning rates varied between the two aviaries. The estimated parameter value gives the differences in rates (i.e. difference in intercept of the baseline or asocial learning rate). We also included both additive and multiplicative models in our model-averaging procedure. Additive models assume that individuals acquire the novel trait as a direct consequence of observation, while multiplicative models assume that social learning occurs indirectly, e.g. by knowledgeable individuals attracting naïve ones to the novel foraging task, where the latter then learn from their own experience. Multiplicative models therefore use the aviary asocial parameter as a variation both

in the intercept of the learning rate over time as well as the slope due to the increase in social learning over time across the two aviaries. Our models are given by the following equations:

$$\lambda_i(t) = \lambda_0(t)\left(\sum_{k=1}^{M}\left[s_k \sum_{j=1}^{N} a_{ijk} z_j(t)\right] + exp(LP_i)\right)\big(1 - z_i(t)\big)$$

for additive, and:

$$\lambda_i(t) = \lambda_0(t)\left(\sum_{k=1}^{M}\left[s_k \sum_{j=1}^{N} a_{ijk} z_j(t)\right] + 1\right) exp(LP_i)\big(1 - z_i(t)\big)$$

for multiplicative models, where $exp(LP_i)$ is set to 0 in models without the asocial parameter included (see *model averaging* below). Because we found very little support for multiplicative models (<0.01% of the Akaike weight), we only included the results of these in the supplemental results (Tables S4 & S5).

We also included models testing for asocial-only learning (all edges in all networks were set to 0) and for undifferentiated social learning (all edges in all networks were set to 1). When testing hypotheses, such as whether individuals learnt only from adults and/or from juveniles, we summed the Akaike weights of all models in each category. All analyses were conducted in R, and model parameters were fitted to the data using the optim function.

Model averaging

We used model averaging to answer two questions: 1) Which are the major pathways of information transfer? and 2) Does information transmission differ through different social ties? We first constructed every possible model with all combinations of the following factors:

- Social transmission model (additive or multiplicative)
- Baseline, or asocial, learning rate (constant or non-constant)
- Network type (foraging-association or homogeneous)
- Asocial parameters (effect of aviary or no asocial parameter)
- Constrained rates (transmission from all individuals, from adults only, or from juveniles only) (used to evaluate question 1)
- Varying transmission rates (same *s* or different *s*) (used to evaluate question 2)

This resulted in 96 unique models, each of which was fitted to the data. In addition, we included 4 models of asocial transmission (with variants for additive/multiplicative, constant/non-constant), in which all *s* values were constrained to 0.

Using log-likelihood of the fit of the model to the data, we calculated the AIC values for each model, and ranked models based on the AICc values. From the resulting Δ AIC values, we then calculated the relative support ($e^{-0.5*\Delta AIC}$). The Akaike weight for that model was the relative support divided by the sum of the relative support for all models.

To measure the Akaike support for each hypothesis, we summed the Akaike weights for all models containing that hypothesis. For example, the support for foraging association versus homogeneous networks is the sum of the weight of the models where the input networks were the observed foraging associations, divided by the total sum of Akaike weights (shown as the

row sums in Table 1). In Figure S1, we show the different models used to evaluate question 1 (all, adult only, or juvenile only networks) and question 2 (same *s* vs different *s*). These 6 combinations were fit with each combination of social transmission model (additive vs multiplicative), baseline learning rate (constant vs non-constant), network type (association vs homogeneous), and asocial parameters (aviary effect vs no aviary effect). Support for same *s* versus different *s* was calculated as the sum of the weights of the 48 models containing same *s* versus the sum of the weights of the 48 models containing different *s*. Similarly, network type consisted of 32 models for each different possible combination of network (all, adults only, juveniles only).

SUPPLEMENTAL REFERENCES

S1. Psorakis, I., Roberts, S.J., Rezek, I., and Sheldon, B.C. (2012). Inferring social network structure in ecological systems from spatio-temporal data streams. J R Soc Interface *9*, 3055-3066.

S2. Psorakis, I., Voelkl, B., Garroway, C.J., Radersma, R., Aplin, L.M., Crates, R.A., Culina, A., Farine, D.R., Firth, J.A., Hinde, C.A., et al. (2015). Inferring social structure from temporal data. Behav Ecol Sociobiol *69*, 857-866.

S3. Farine, D.R. (2013). Animal Social Network Inference and Permutations for Ecologists in R using asnipe. Methods Ecol Evol *4*, 1187–1194.

S4. Franz, M., and Nunn, C.L. (2009). Network-based diffusion analysis: a new method for detecting social learning. P Roy Soc B-Biol Sci *276*, 1829-1836.

S5. Hoppitt, W., and Laland, K.N. (2011). Detecting Social Learning Using Networks: A Users Guide. Am J Primatol *73*, 834-844.

S6. Hoppitt, W., Boogert, N.J., and Laland, K.N. (2010). Detecting social transmission in networks. J Theor Biol *263*, 544-555.

S7. Farine, D.R., Aplin, L.M., Sheldon, B.C., and Hoppitt, W. (2015). Interspecific social networks promote information transmission in wild songbirds. P Roy Soc B-Biol Sci *282*, 20142804.
